# Exploring perceptions, knowledge, and attitudes regarding pharmacogenetic testing in the medically underserved

**DOI:** 10.3389/fgene.2022.1085994

**Published:** 2023-01-13

**Authors:** Brian E. Gawronski, Emily J. Cicali, Caitrin W. McDonough, Linda B. Cottler, Julio D. Duarte

**Affiliations:** ^1^ Department of Pharmacotherapy and Translational Research, College of Pharmacy, University of Florida, Gainesville, FL, United States; ^2^ Center for Pharmacogenomics and Precision Medicine, College of Pharmacy, University of Florida, Gainesville, FL, United States; ^3^ Department of Epidemiology, College of Public Health and Health Professions and College of Medicine, University of Florida, Gainesville, FL, United States

**Keywords:** pharmacogenetic, underserved, survey, attitudes, implementation

## Abstract

**Introduction:** Pharmacogenetic testing may hold promise in addressing health disparities, as medically underserved patients appear to be prescribed medications with pharmacogenetic guidelines at higher rates. While routine clinical implementation of testing in medically underserved populations has not yet been achieved, using patient perspectives to inform implementation should increase the likelihood of success. The aim of this study was to assess the perceptions, knowledge, and attitudes regarding pharmacogenetic testing in medically underserved patients.

**Methods:** We developed a survey instrument to assess respondent views on pharmacogenetic testing. The survey instrument was developed through a process of literature review, expert input, iterative pilot testing, and final refinement. The survey instrument was fielded to US adults with an estimated household income of $42,000 per year or less.

**Results:** During the survey instrument development, 59 pilot testers provided 133 comments which lead to 38 revisions to the survey instrument. The nationwide survey resulted in 1,060 respondents, of which half (49.8%) reported having no health insurance or being on Medicaid. Most patients (78.9%) had not previously heard of pharmacogenetic testing. After being provided an explanation of pharmacogenetic testing, 60.5% were very or moderately interested in receiving testing if there were no cost and 75.8% of respondents agreed or strongly agreed that pharmacogenetic testing should be available to help with medication selection regardless of cost. Respondents shared that their greatest concern with pharmacogenetic testing was that the test would cost them money, which was expressed by over half (52.7%). This was followed by concerns that the results could reveal a risk for a disease, could affect health insurance, and would not improve care.

**Discussion:** Our results indicate a strong interest in pharmacogenetic testing and identify key perceptions, attitudes, concerns, and potential barriers that can be addressed as pharmacogenetic testing is clinically implemented in medically underserved patient populations.

## 1 Introduction

Precision medicine has the potential to improve health outcomes by factoring individual patient lifestyle, environmental and genetic variability into treatment decisions (Denny and Collins, 2021). Pharmacogenomics, one aspect of precision medicine, assesses the impact of genetic variability on patient drug response. Clinical implementation of pharmacogenetic testing is emerging in an environment of existing health disparities in the United States (Centers for Disease Control and Prevention, 2013; Dunnenberger et al., 2015; Chetty et al., 2016; Anderson and Zimmerman, 2021). In part due to the lack of widespread clinical implementation of pharmacogenetics, there is a paucity of research on the impact of pharmacogenetics on health disparities with current evidence supporting both potential reductions and increases in health disparities (Martin et al., 2017).

The underrepresentation of minority groups in pharmacogenetics research has raised concerns for equity (Sirugo et al., 2019). There is additional concern that the implementation of pharmacogenetic testing has the potential to exacerbate health disparities due the inverse equity hypothesis. The inverse equity hypothesis states that as a new health technology is implemented it is first accessible to populations with higher socioeconomic status with delayed accessibility for populations with lower socioeconomic status, who are often at greatest need. This leads to greater health disparities (Victora et al., 2000). Medically underserved patients can be most impacted by these disparities as they are often the last to experience the benefits of the implementation of health technology and face barriers to accessing healthcare due to geographic and socioeconomic factors (Weiss et al., 2018).

Clinical implementation of pharmacogenetic testing is poised to follow the inverse equity hypothesis, especially in medically underserved populations. The majority of pharmacogenetic implementations have occurred in academic medical centers, which limits access for those not living near these institutions. The lack of widespread insurance coverage for testing (Park et al., 2020), notwithstanding recent changes in Medicare coverage (Centers for Medicare & Medicaid Services, 2021) also limits access. Medically underserved patients are uniquely poised to receive benefit from pharmacogenetic testing due to higher utilization of off-patent generic drugs, which have pharmacogenetic guidelines, and the potential for a reduction of healthcare visits required for medication optimization (Duconge and Ruaño, 2012). Additionally, medically underserved patients are prescribed medications with pharmacogenetic guidelines available at higher rates than other populations (Dalton et al., 2021). Despite the risk for an increase in health disparity, the potential to ameliorate the risk through the deliberate implementation of pharmacogenetic testing in the medically underserved exists. However, a pivotal challenge in the implementation of pharmacogenetic testing is engaging patients to participate and utilize testing through understanding patient knowledge, attitudes, and perceptions.

Previous studies have evaluated various patient populations for their knowledge, attitudes, and perceptions towards pharmacogenetic testing (Rogausch et al., 2006; Zhang et al., 2014; Lachance et al., 2015; Chapdelaine et al., 2021; Kastrinos et al., 2021; Allen et al., 2022; Martin et al., 2022). However, there has been limited evaluation of the views of medically underserved populations. Thus, the aims of this study were to develop a survey instrument and to the utilize this instrument to assess the perceptions, knowledge, and attitudes regarding pharmacogenetic testing in a nationwide survey of a primarily medically underserved population with the hope that these insights can be utilized to guide the implementation of pharmacogenetic testing in this patient population.

## 2 Materials and methods

### 2.1 Survey instrument development

A survey instrument was developed to assess perceptions, knowledge, and attitudes regarding pharmacogenetic testing. This survey instrument was specifically developed for use in a clinical setting to be administered before, and then after, pharmacogenetic testing. The initial domains of the survey were determined through an iterative process of literature review and expert input. Prior studies and patient surveys that evaluated genetic testing, pharmacogenetic testing, and clinical implementation of genomic medicine were examined (Haga et al., 2013; Henneman et al., 2013; Weitzel et al., 2016; Lee et al., 2018; Orlando et al., 2018). Based on the literature review, questions were organized into eight domains: demographics, general health, previous adverse reactions from medications, knowledge, attitudes, payment, sharing, and satisfaction (Supplementary Table S1). Most of the questions (∼80%) were based on the literature with extensive modification for applicability for pharmacogenetics with the balance of the questions developed by individuals with expertise in pharmacogenetics. Following initial instrument drafting, additional revision was conducted by expert input, focusing on pharmacogenetics and overall best practices in patient surveys. Additional questions were drafted as a part of the expert review to add clarification and to expand the surveyed items in the domains. Additionally, the questions were edited for readability.

The survey instrument then underwent an iterative process of pilot testing and modifications based on the feedback from the pilot testers. In addition to the survey instrument, pilot testers were asked pilot questions regarding feasibility, ease of completing, understandability, medical jargon, and length. The pilot testers included epidemiology graduate students, pharmacogenetics graduate students, pharmacogenetics post-doctoral fellows, and non-expert community members who were part of a community outreach program, HealthStreet, based out of the University of Florida Clinical and Translational Science Institute (Serdarevic et al., 2017). Pilot testers were able to provide feedback for the overall survey and by question, to identify areas that needed modification. The feedback from the pilot test was grouped into the following themes: understandability or clarity of the question, understandability or clarity of the answer(s), formatting or grammar of the question, formatting or grammar of answer, and testing mechanics.

The domains and questions were finalized based on feedback from the iterative pilot testing process. The final survey was adapted to a data dictionary that can be easily uploaded into Research Electronic Data Capture (REDCap) (Harris et al., 2019) or other online survey tools. The final survey questions (Supplementary Table S2) as well as the data dictionary are included in the Supplementary Material. For utilization of the survey instrument in the nationwide, single time point survey of medically underserved individuals, questions were further compiled, arranged, and edited. The survey utilized in the nationwide survey can be found in Supplementary Material. The survey was developed for online and mobile administration utilizing the Qualtrics platform, version 09/2022 (Qualtrics, Provo, UT).

### 2.2 Nationwide survey population

Between 29 March 2022 and 19 April 2022, an online convenience sample was collected utilizing Qualtrics Research Services (Qualtrics, Provo, UT). To be eligible to complete the survey, respondents were 18 years of age or older, resided in the United States, and had an average yearly household income of $42,000 or less. In order to survey a population of likely medically underserved respondents, $42,000 represents 150% of the 2021 United States Census Bureau poverty threshold for a household of four, which is $27,949 (United States Census Bureau, 2022), as the average family household size is 3.21 per the 2021 United States Census Bureau Average Number of People per Household table (United States Census Bureau, 2021a). Respondents were sourced from Qualtrics panels of proprietary survey respondents. This study was approved by the University of Florida Institutional Review Board. Respondents who were screened eligible were provided a cover letter prior to starting the survey outlining their rights as respondents and stating that respondents were free to end participation in the survey at any time.

### 2.3 Survey methods

Recruitment, participant sampling, and administration of the survey online or on a mobile device was completed by Qualtrics. The survey remained open until 1,100 respondents with completed surveys was reached with oversampling conducted to cover removal of low-quality responses. Quality control was conducted on the survey responses following survey collection. Surveys which were initiated but not completed by the end of the collection period and responses which represented speeders, defined as completing the survey in less than 2 min and 30 s, were removed. Responses with flatline answers, duplicate or responses from the same household, logically discordant answers, invalid zip codes, invalid ages, and those who indicated invalid household incomes were also removed. An attention check question was included in the survey to assess participant engagement and understanding, and responses were not included if this question was not appropriately answered (Abbey and Meloy, 2017). Health literacy was assessed utilizing a slider on a scale from 0 to 100 where 0 indicated not health literate and 100 indicated very health literate. Attitudes and perceptions regarding pharmacogenetic testing were accessed with questions utilizing a Likert scale where five corresponded to strongly agree and one to strongly disagree. After answering demographic questions, respondents were provided an explanation of pharmacogenetic testing (included in the Supplementary Material). Questions assessing the attitudes and opinions regarding pharmacogenetic testing were presented to respondents in a randomized order. Zip codes were utilized to map over to Zip Code Tabulation Areas (ZCTA) utilizing the UDS Mapper zip code to ZCTA cross walk for 2021 (Health Resources and Services Administration, 2021) which were then mapped over to Social Deprivation Index (SDI) 2015 values from the Robert Graham Center calculated as previously described (Butler et al., 2013).

### 2.4 Statistical analysis

Demographics of the pilot testers and pilot tester responses to the pilot questions were summarized utilizing mean and standard deviation (SD) for continuous variables and counts and percentages for categorical variables. Summary statistics and analysis for survey instrument development and pilot testing were conducted using SAS v 9.4 (SAS Institute Inc., Cary, NC, United States). The demographics for the nationwide survey cohort were summarized with median and interquartile range (IQR) for continuous variables, due to a lack of normality in distribution, and counts and percentages for categorical variables. For survey measures including pharmacogenetic knowledge and interest, attitudes, and perceptions, and sharing and pharmacogenetic results, subgroup analysis was performed assessing self-reported race, Hispanic ethnicity, and SDI scores. SDI was categorized into quartiles with quartile 1 representing respondents with the lowest level of disadvantage while quartile 4 the highest level. For continuous variables, subgroups were analyzed utilizing the Kruskal Wallis Test with *post hoc* pairwise Wilcoxon Rank Sum Tests with adjustment for multiple comparison by False Discovery Rate for subgroups with more than two levels. For categorical data, subgroups were analyzed utilizing Chi-squared tests and Fisher’s exact tests. The survey data were analyzed utilizing R version 4.1.1 (R Core Team, 2022).

## 3 Results

### 3.1 Survey development and pilot testing

The initial survey instrument contained 53 questions between the pre-pharmacogenetic testing and post-pharmacogenetic testing surveys, which was increased to a total of 74 questions after expert review. A total of 59 pilot testers reviewed the survey, however not all testers answered or provided feedback on all questions. The average age of the pilot testers was 35.78 ± 15.6 (n = 51, mean ± SD), and the majority (60%, 31/52) were male. Of the 50 pilot testers who provided their highest level of education achieved, 28% had a high school diploma equivalent or less, 8% had an associate degree, 28% had a bachelor’s degree, and 36% had a graduate degree. Pilot testers provided 133 comments on individual questions which were categorized into prespecified themes, where the largest category (32%) was “clarity of questions” (Supplementary Figure S1). Based on the comments and feedback, 38 revisions were identified, largely attributable to modifying and improving the clarity of the question (43%) (Supplementary Figure S2). The results from the pilot questions indicated that the survey would be feasible to administer regardless of location. Overall, pilot testers indicated that the survey was easy to take, and the questions were understandable, with little medical jargon. Nearly three quarters felt that the length of survey was just right (Supplementary Table S3).

### 3.2 Survey respondents, demographics, and general health

**FIGURE 1:**
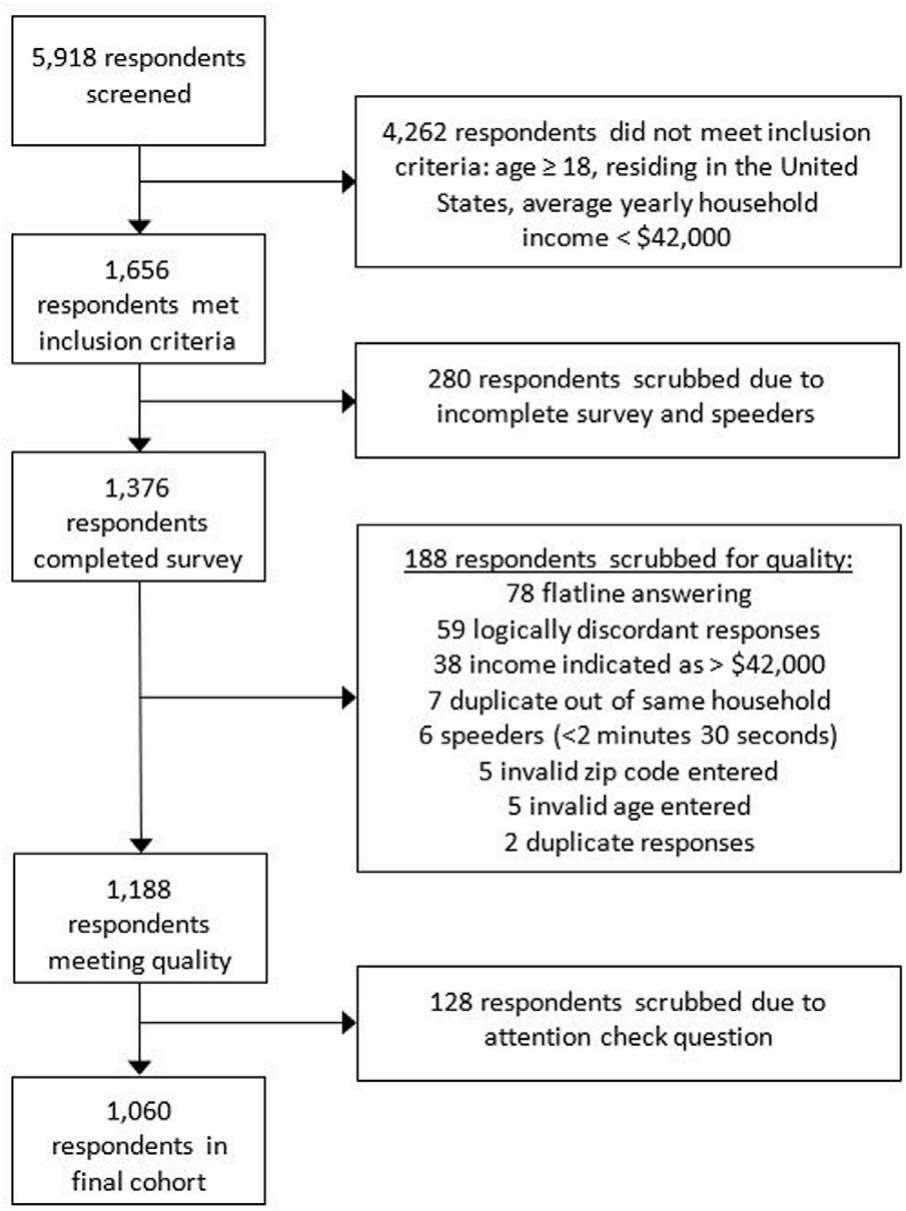
Nationwide survey respondent flow chart and quality control details for exclusion of respondents. The reasons for respondents scrubbed for quality will total to more than 188 as some respondents were scrubbed for multiple reasons.

For the nationwide survey, a total of 5,918 respondents were screened for the inclusion criteria of which 1,656 respondents, or 27.9% met the inclusion criteria. 1,376 respondents completed the survey representing an 83.1% completion rate. After quality control, 316 respondents were removed resulting in a final cohort of 1,060 respondents (Figure 1). Respondents completed the survey in a median of 7 min and 34 s (IQR: 4 min and 34 s).

The demographic characteristics of the respondents are summarized in Table 1. The respondents had a median age of 42 years of age, were 67% female, and had a median SDI score of 60.5. The respondents resided in 48 States, with only Alaska and South Dakota lacking respondents (Supplementary Figure S3). For self-reported race, 13.6% identified as African American/Black, 3.5% as Asian, and 6.2% as mixed race, another race, American Indian/Alaskan Native, or Pacific Islander/Native Hawaiian. Of the respondents, 11.7% self-identified as Hispanic, Latino, or Latinx. The majority of respondents (58.1%) indicated their highest level of education was a high school diploma equivalent or less and many respondents (45.2%) worked full- or part-time jobs. Only 27.2% of respondents indicated they have enough money to purchase additional things they wanted after paying the bills and 14.6% had trouble paying the bills no matter what they did.

**TABLE 1:** Respondent demographics (*n* = 1,060)^a^.

Age, years (median (IQR))	—	42 (25)
Social deprivation index (median (IQR))	—	60.5 (40)
Gender	Female	711 (67.1)
	Male	343 (32.4)
	Other	6 (.6)
Self-reported race	Caucasian/White	814 (76.8)
	Black/African American	144 (13.6)
	Asian	37 (3.5)
	Another Race	25 (2.4)
	Mixed Race	23 (2.2)
	American Indian/Alaskan Native	16 (1.5)
	Pacific Islander/Native Hawaiian	1 (.1)
Hispanic, Latino, Latinx	Yes	124 (11.7)
	No	931 (87.8)
	Did not know	5 (.5)
Highest level of education/Training achieved	Elementary or junior high	37 (3.5)
	High school diploma	503 (47.5)
	GED	76 (7.2)
	Vocational tech diploma	93 (8.8)
	Associate degree	162 (15.3)
	Bachelor’s degree	156 (14.7)
	Master’s degree	25 (2.4)
	Doctorate or professional degree	8 (.8)
Income	Less than $17,500	289 (27.3)
	$17,500 to $26,500	280 (26.4)
	$26,600 to $35,500	268 (25.3)
	$35,600 to $42,000	223 (21.0)
Occupation	Working full-time	307 (29.0)
	Working part-time	172 (16.2)
	Full-time homemaker	96 (9.1)
	Disability	108 (10.2)
	Retired	195 (18.4)
	Unemployed of laid off Something else	144 (13.6)
		38 (3.6)
Number of people in household	1 or 2	614 (57.9)
	3 or 4	323 (30.5)
	5 or more	123 (11.6)
Work in Health or Biomedical Field	Yes	169 (15.9)
	No	882 (83.2)
	Did not know	9 (.8)

^a^ Demographics are summarized as mean (SD) unless otherwise specified.

Regarding health literacy, general health, and health insurance of the respondents, respondents reported a median health literacy score of 71.5 (IQR: 31). In rating their general health, 30% of respondents indicated they were in excellent or very good health, while 29% indicated they were in fair or poor health. Most respondents (64%) agreed or strongly agreed that there was something available that could improve their health. About half of respondents reported not having health insurance (15.9%) or being on Medicaid (33.9%). The remaining respondents reported being insured through commercial insurance (20%), Medicare (25.9%), and other governmental insurance (4.3%).

### 3.3 Pharmacogenetics knowledge and interest

**FIGURE 2:**
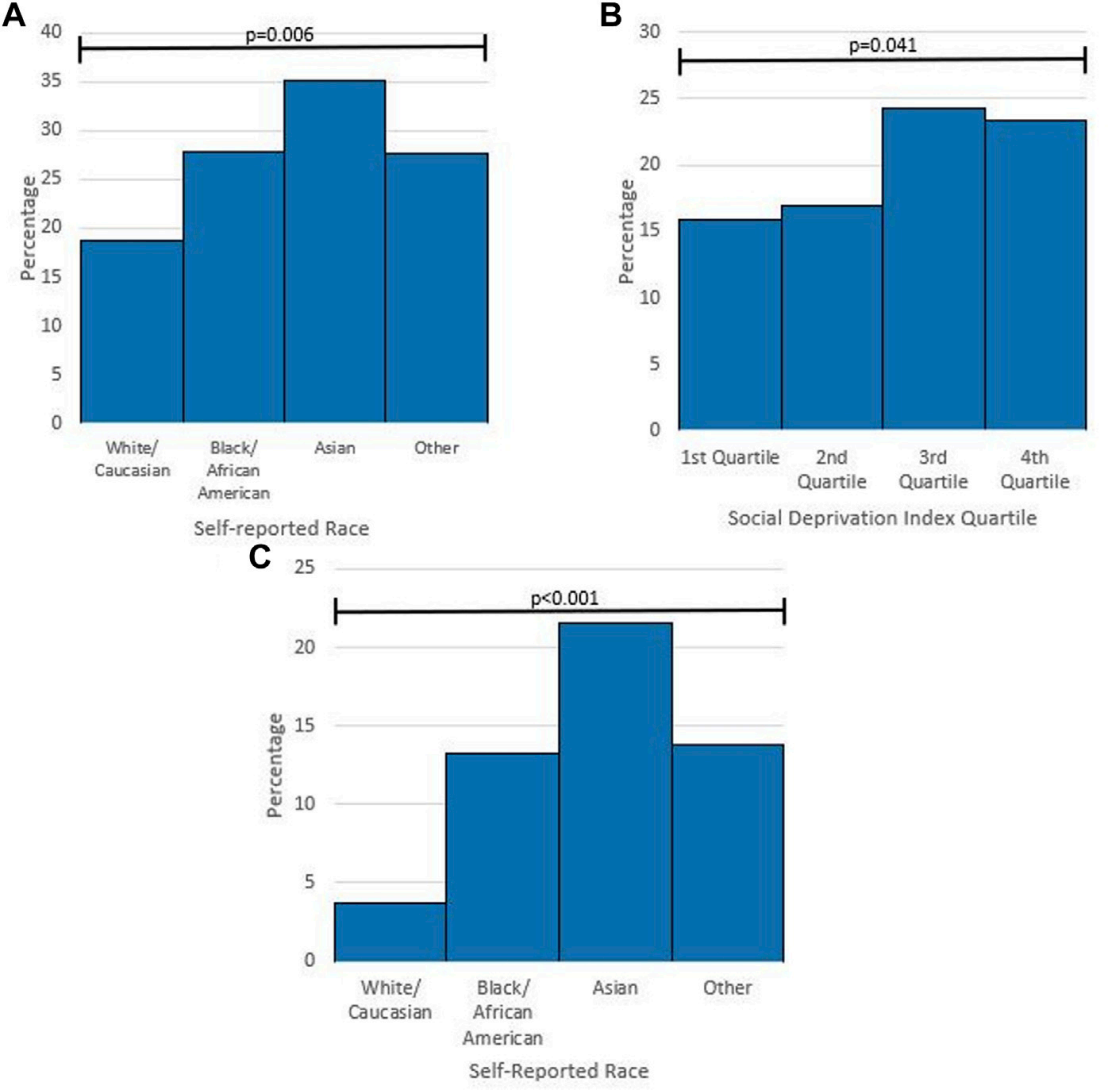
Subgroup analysis of pharmacogenetic knowledge and previous pharmacogenetic testing **(A)** percentage of respondents who had previously heard of pharmacogenetic testing by self-reported race. *p*-value from Chi-square Test. **(B)** Percentage of respondents who had previously heard of pharmacogenetic testing by Social Deprivation Index Quartile. *p*-value from Chi-square Test. **(C)** Percentage of respondents who had previous pharmacogenetic testing by self-reported race. *p*-value from Fisher Exact Test.

**FIGURE 3:**
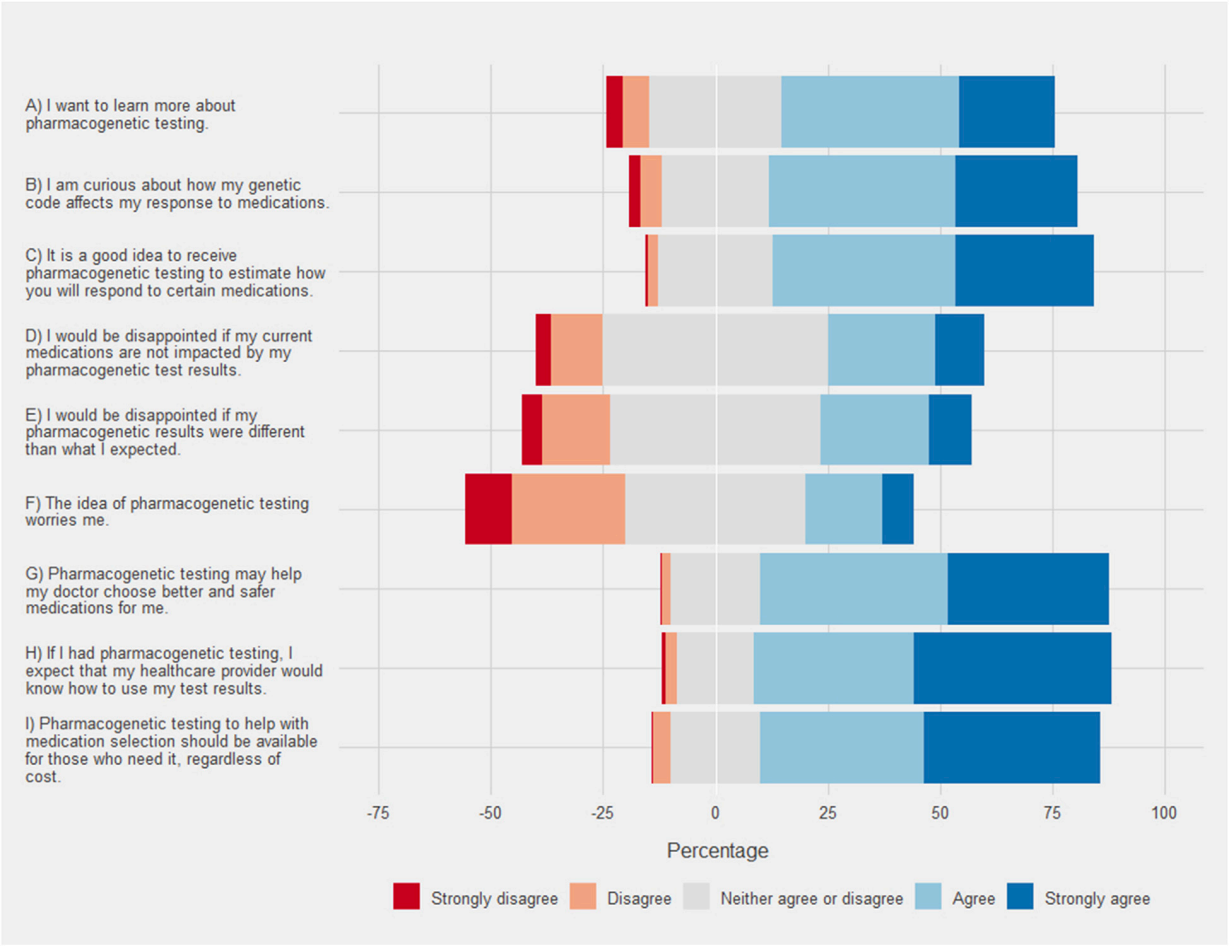
Respondent attitude and perceptions of pharmacogenetic testing. Each bar corresponds to the percentage of each group on a Likert scale where the neutral response is centered on zero. Agreement is show in positive percentages (light blue and blue) and disagreement is shown as negative percentages (light red and red).

Prior to the explanation of pharmacogenetic testing included as part of the survey, only 21.1% of respondents had previously heard of pharmacogenetic testing. This was significantly different between self-identified race groups and SDI quartiles. For self-identified race, White/Caucasian respondents showed lower rates of prior knowledge of pharmacogenetic testing, while Asians showed higher rates when compared with African American/Black respondents and respondents from other race groups (Figure 2A). In addition, there were significantly different levels of previous knowledge of pharmacogenetic testing between SDI quartiles (Figure 2B). Only 6.2% of respondents were aware of previously receiving a pharmacogenetic test. For self-identified race, 3.7% (30/814) of White/Caucasian respondents reported previous pharmacogenetic testing, 13.2% (19/144) of African American/Black respondents, 21.6% (8/37) of Asian respondents, and 13.8% (9/65) of respondents from other race groups (*p* < .001) (Figure 2C). Following the explanation of pharmacogenetic testing included in the survey, 60.6% expressed high or moderate interest in receiving pharmacogenetic testing if it were offered to them at no cost (Figure 3).

### 3.4 Attitudes and perceptions

**FIGURE 4:**
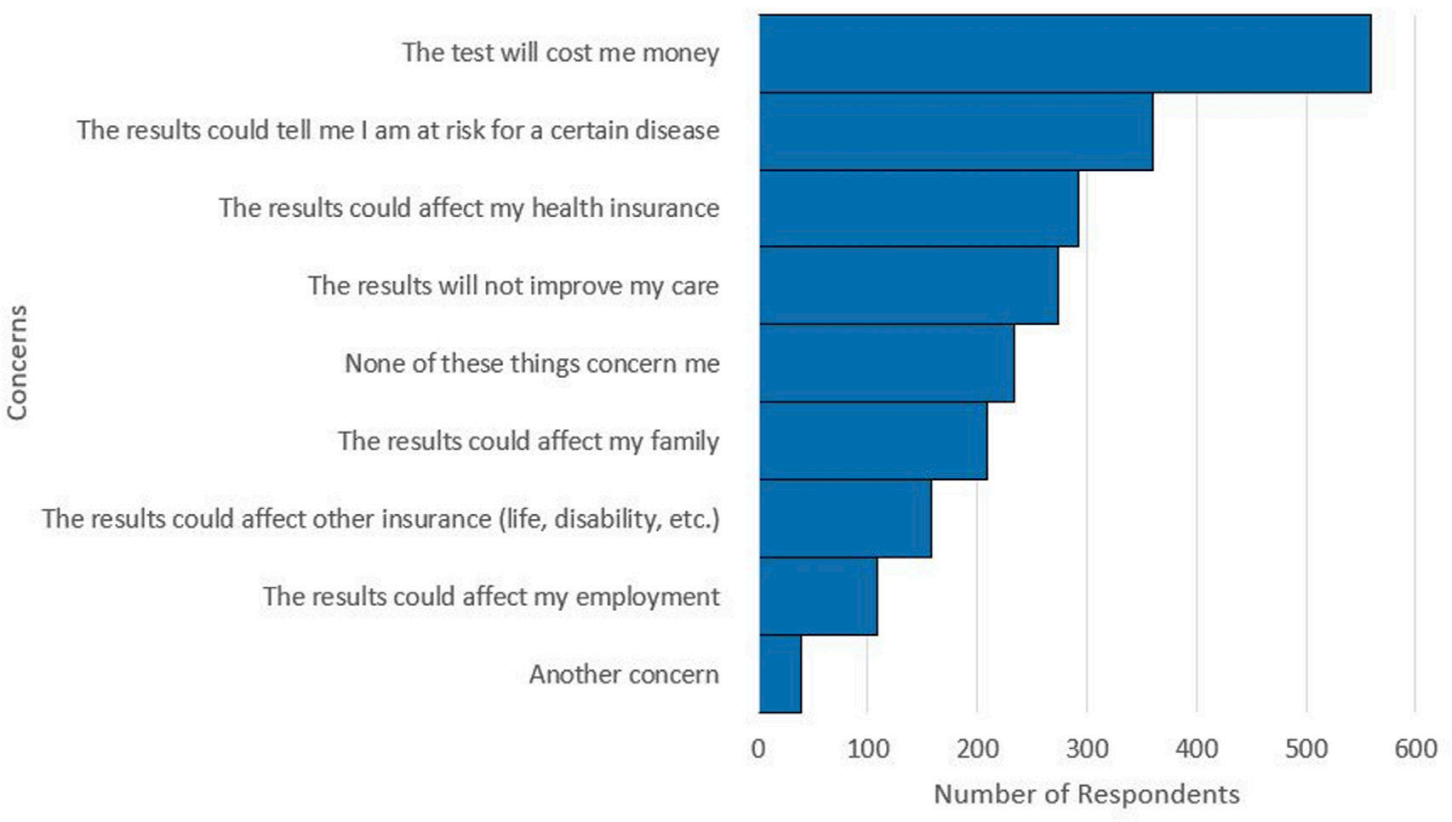
Respondent selections for concerns with pharmacogenetic testing. Multiple concerns could be selected by respondents. A total of 2,234 concerns were selected by the 1,060 respondents.

Respondents overwhelmingly indicated that they agreed or strongly agreed that pharmacogenetic testing may help their doctor choose better or safer medications for them (Figure 4). The respondent results were more evenly distributed between agreement and disagreement with regard to potential disappointments. Only, 33.7% of respondents agreed or strongly agreed that they would be disappointed if their pharmacogenetic results were different from what they expected. Black/African American respondents agreed to a greater extent that they would be disappointed (mean 2.06, SD 0.96) when compared to White/Caucasian respondents (mean 1.79, SD 1.05 [*p* < .001]). Similarly, Hispanic respondents also agreed to a greater extent that they would be disappointed (mean 2.54, SD 0.96) when compared to non-Hispanic respondents (mean 2.15, SD .95 [*p* < .001]). Additionally, the highest SDI quartile agreed that they would be disappointed to a greater extent (mean 2.31, SD 1.02) when compared to the other quartiles (mean 2.14, SD .93 [*p* = .003]). Some respondents (24.2%) agreed or strongly agreed that pharmacogenetic testing worried them. Black/African American respondents agreed to a greater extent that they were worried about pharmacogenetic testing (mean 2.51, SD .91) compared to White/Caucasian respondents (mean 2.12, SD .95 [*p* = .011]). Similar results were obtained when comparing Hispanic respondents (mean 2.1, SD 1.14) to non-Hispanic respondents (mean 1.82, SD 1.04 [*p* = .008]). Finally, the strongest agreement was seen with many respondents (79.6%) expecting that their healthcare provider would know how to use their test results.

Respondents also shared their concerns regarding pharmacogenetic testing. Of the 1,060 respondents, 22.1% indicated that they had no concerns with pharmacogenetic testing. The most frequently selected concern was that the test would cost the respondent money, which was selected by over half of the respondents (52.7%). Cost of pharmacogenetic testing was the most selected concern, regardless of self-reported race group, ethnicity group, or SDI quartile. The next most selected concerns in order of frequency were: the results could reveal a risk for a disease, the results could affect health insurance, and the results would not improve care (Figure 4).

### 3.5 Sharing and pharmacogenetic results

Most respondents (84.3%, 634/752) indicated they would share their pharmacogenetic results with their spouse/partner, while 70.5% (540/766) would share with their parents, 67.3% (512/761) with their children, 65.1% (571/877) with their siblings, and only 49.0% (435/887) with other family members. A majority of respondents (65.2%, 609/934) would also share their results with a pharmacist.

**FIGURE 5:**
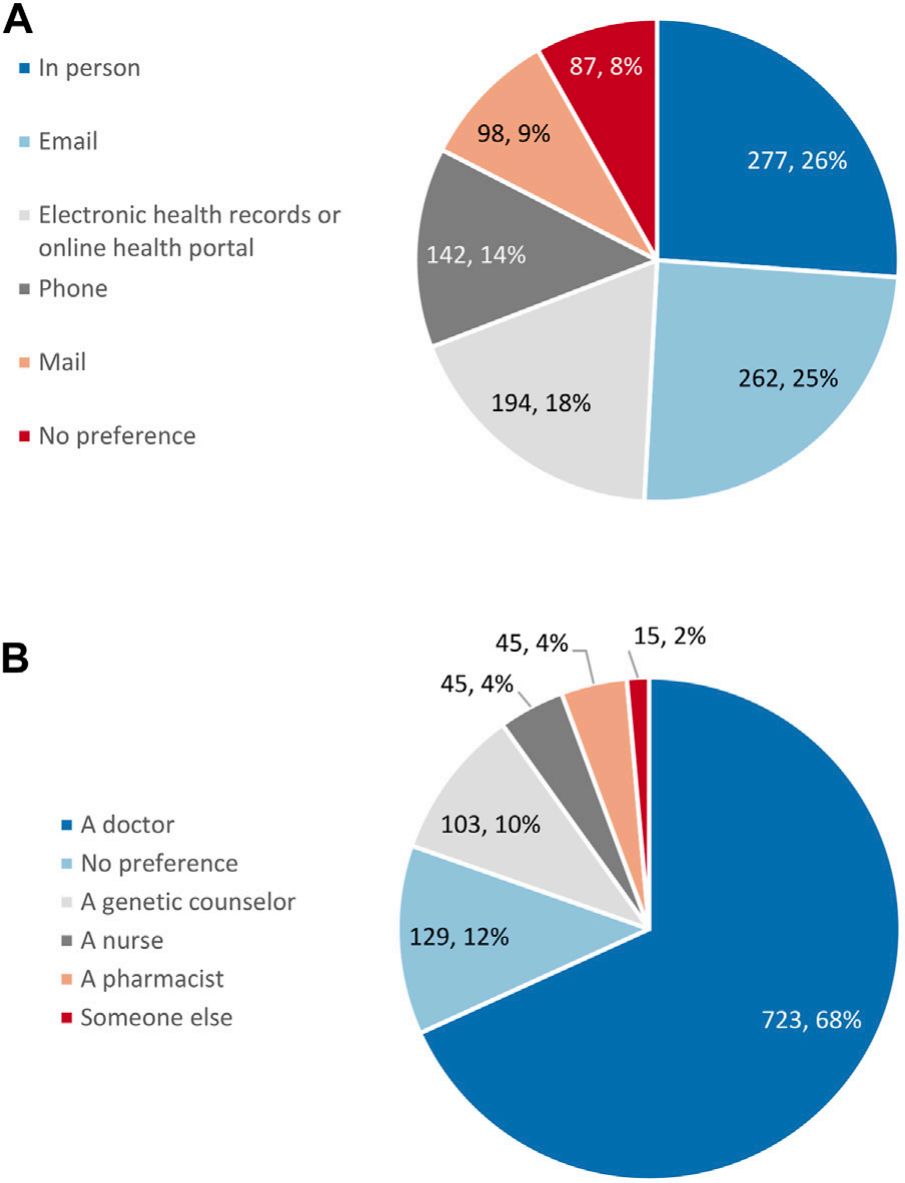
Respondent preference for **(A)** the method for the return of pharmacogenetic results (*n* = 1,060) and **(B)** who would explain pharmacogenetic results (*n* = 1,060).

Respondent preference on the method they received their results was widely distributed. Receiving results in person, by email, and through the electronic health record were most popular, while receiving results by phone and through the mail were the least preferred (Figure 5A). A similar proportion of respondents (43%) preferred digital methods for receiving results (email and the electronic health record) as those respondents (49%) who preferred analogue methods (in person, by phone, and by mail). Respondents, by a large margin, preferred that a doctor would explain their pharmacogenetic test results to them (Figure 5B).

## 4 Discussion

In this study, we created a survey instrument that was developed and revised through an iterative process including literature review, expert input, and pilot testing in the community. The survey instrument can be utilized clinically for patients before and after undergoing pharmacogenetic testing, however the survey instrument can be modified for use in other settings. We demonstrate this fact by using it in a first-of-its-kind nationwide evaluation of the knowledge, attitudes, and perceptions towards pharmacogenetic testing in primarily medically underserved respondents. The survey instrument has been made publicly available (see Supplementary Materials) to facilitate future research by others in the field to provide more robust data on the perspectives toward pharmacogenetic testing of diverse populations.

Medically underserved respondents in the nationwide survey were broadly supportive and receptive toward pharmacogenetic testing. A large majority of respondents indicated at least some level of interest in pharmacogenetic testing, which is promising for utilization and uptake in future implementation efforts in this patient population. Most respondents agreed or strongly agreed that they wanted to learn more about pharmacogenetic testing, were curious about how their genetic code would affect their response to medications, and that pharmacogenetic testing could help their doctor with medication choice. This is consistent with the findings in other patient populations (Trinidad et al., 2015; Allen et al., 2022). Our data also indicated that patient testing expectations could be a potential barrier to patient acceptance of pharmacogenetic test results. This is especially true for Black/African American and Hispanic respondents. These findings align with differences in the concerns regarding personalized medicine by race which have previously been reported (De Marco et al., 2010). Likewise, differences between SDI quartiles and potential disappointment with pharmacogenetic test results was also noted. Pharmacogenetic testing implementation in medically underserved patient populations will need to ensure pre-test materials and counseling are available to manage testing expectations, especially in Black/African American, Hispanic, and lower socioeconomic status medically underserved populations.

Respondent worries and concerns quantified in this study will be important potential barriers that will need to be addressed in future pharmacogenetic testing implementation. The cost of pharmacogenetic testing was the most frequently indicated concern. While cost is an important factor in the implementation of any new technology, medically underserved populations are particularly susceptible to cost as a barrier for healthcare (Virapongse and Misky, 2018). Studies have previously assessed the willingness to pay for pharmacogenetic testing (Bielinski et al., 2017; Regier et al., 2020). However, additional study of what medically underserved populations are willing to pay for pharmacogenetic testing is needed to aid in guiding implementation efforts and the design of pharmacogenetic testing panels with costs that are accessible to this patient population.

Respondents were also concerned that their results would affect their families, affect health insurance or other insurance such as life insurance, reveal risk of a disease, and affect their employment. Patient education materials and resources would need to be crafted to address and alleviate these concerns to improve uptake (Mills et al., 2017; Mills and Haga, 2018; Asiedu et al., 2020). Ensuring success of implementation of pharmacogenetic testing in medically underserved populations will rely on involving patients prior to and during implementation to assist not only in designing patient facing communication, but also implementation strategies and approaches (Rosenman et al., 2017; Sperber et al., 2017). Understanding and appreciating patients’ preconceived ideas and concerns is key to designing effective implementation of pharmacogenetic testing programs (Rosenman et al., 2017).

Our results provide important insights into how medically underserved respondents prefer to receive their pharmacogenetic results. Based upon the broad distribution of preferences for the method by which results are received, implementation efforts should ensure that there are multiple avenues for result dissemination. Digital options such as email and use of the electronic health record were preferred in similar proportion by respondents as analogue methods such as in person, by phone, or by mail. However, the electronic delivery of our survey instrument could have potentially introduced bias toward digital option preference. Previous research has shown that Internet access is not a primary barrier in medically underserved populations and as such should not be ruled out as an accessible method for disseminating results in this population (Zach et al., 2012). However, recent research has indicated certain patient groups such as those over the age of 65, Black/African American, and Hispanic/Latino patients are more likely to utilize in-person visits over telehealth visits (Weber et al., 2020). Thus, a combination of digital and analogue methods for return of results should be considered. The medically underserved respondents overwhelmingly preferred that a doctor explain their pharmacogenetic results. Despite this preference, 76% of primary care physicians are uncomfortable with applying pharmacogenetic test results to their prescribing (Olander et al., 2018). Healthcare providers indicate that pharmacists should play a major role in implementing pharmacogenetic test results in clinical practice (Frigon et al., 2019), while pharmacists were one of the least preferred to explain results in the current study. Implementation of pharmacogenetic testing in medically underserved patients will have to bridge this preference divide, ensure adequate training and resources are available for primary care providers, and look to ensure coordination between pharmacists and primary care providers.

Despite the several strengths our study possesses, there are also important limitations to consider. Due to the convenience sampling methods utilized by the survey, the possibility for sampling bias and a lack of generalizability of the results is present. Our results indicate, however, based on the demographic characteristics (Table 1) that the sample that was surveyed closely matched the general United States population (United States Census Bureau, 2021b) with regards to age and self-reported race. Key differences to note are the overrepresentation of female respondents, which can be expected given females are more likely to participate in surveys compared to males (Becker, 2022), and underrepresentation of respondents identifying as Hispanic, Latino, or Latinx in our sample compared to the United States population. Our survey instrument was only developed and administered in English, which could have contributed to the underrepresentation of this population, as well as other medically underserved populations whose preferred language is not English. However, this large (n = 1,060) and nationwide study provides the largest sample to date evaluating medically underserved patients filling a current gap in knowledge as there has been limited previous study of the knowledge, attitudes, and perceptions toward pharmacogenetic testing in medically underserved populations (O'Daniel et al., 2010; Lee et al., 2018; Canedo et al., 2019; Stegelmeier et al., 2020; Saulsberry et al., 2021). Our results provide a framework of potential barriers and key attitudes, perceptions, and concerns that medically underserved patients have toward pharmacogenetic testing which can act as a starting point for assessments in specific communities. Our results identify key points that can guide the important process of engagement with key local stakeholders prior to implementation efforts—particularly in settings where the patient population varies significantly from that reported in this study.

The inclusion criteria for our survey were designed to select for medically underserved respondents utilizing a federal poverty limit cut-off. Understanding that medically underserved populations in the United States are defined utilizing additional criteria such as number of primary care physicians per capita and infant mortality rates, this is a potential limitation for our study (Health Resources and Services Administration, 2019). However, income has long been an indicator utilized for defining medically underserved populations (Ricketts et al., 2007) and is well correlated with being medically underserved (Kim et al., 2020). Given the challenges associated with precisely identifying a medically underserved population in an online and mobile based nationwide survey, the characteristics of our sample— lower levels of education, lacking health insurance or being on Medicaid, lower levels of healthcare utilization, economically constrained, and needing an improvement in health— are in line with a medically underserved population. Additionally, the median SDI score in our study is similar to what is noted in other studies of disadvantaged and underserved populations (Patel et al., 2020; Green et al., 2022) and is higher than noted in the assessment of general patient populations (Babatunde et al., 2021; Babatunde et al., 2022).

Medical innovations have the potential to reduce social inequalities (Rydland, 2021). Whether the implementation of pharmacogenetic testing can make an end run around the potential of the inverse equity hypothesis through the early implementation in medically underserved populations remains to be demonstrated. To that end, the results of this study in a medically underserved population identified strong interest in pharmacogenetic testing. The potential barriers and key perceptions, attitudes, and concerns toward pharmacogenetic testing were identified and can inform the future clinical implementation of pharmacogenetic testing in medically underserved patients.

## Data Availability

The raw data supporting the conclusions of this article will be made available by the authors, without undue reservation.
