# Supplementary material for: Exploring perceptions, knowledge, and attitudes regarding pharmacogenetic testing in the medically underserved
Source: Front Genet. 2023 Jan 13;13:1085994. doi: 10.3389/fgene.2022.1085994 (PMC9880414; doi:10.3389/fgene.2022.1085994)
Supplement: Supplementary file 1 [file DataSheet1.zip › Supplementary Materials.docx]

Supplementary Material

# Supplementary Data

Supplementary Figures and Tables

Table S1

Table S2

Table S3

Figure S1

Figure S2

Figure S3

Medically Underserved Nationwide Survey Instrument

Additional Supplementary File:

Pharmacogenetic Testing Survey Data Dictionary.csv

# Supplementary Figures and Tables

## Table S1

| - **Table S1**: Questions and Domains in the Survey Instrument | | | | |
| --- | --- | --- | --- | --- |
| **Domains** | **Initial Survey** | | **Revised and Final Survey** | |
|  | **Pre Survey** | **Post Survey** | **Pre Survey** | **Post Survey** |
|  | Questions^*^, n | | | |
| Demographics | 5 | NA | 7 | 4 |
| General health | 2 | NA | 3 | NA |
| Previous adverse reactions from medications | 2 | NA | 2 | NA |
| Knowledge | 6 | 2 | 7 | 5 |
| Attitudes | 6 | 6 | 6 | 6 |
| Payment | 1 | 1 | 2 | 1 |
| Sharing | 1 | 3 | 9 | 10 |
| Health Literacy | NA | 3 | NA | NA |
| Perceptions | NA | NA | 7 | NA |
| Satisfaction | NA | 5 | NA | 5 |
| **Total Questions** | 23 | 20 | 43 | 31 |

^*^Users may answer less questions pending on their responses to certain questions, as a skip pattern is present

## Table S2

| **Table S2**: Final Survey Questions in Instrument | | | |
| --- | --- | --- | --- |
| **Pre Test** | | **Post Test** | |
| **Number Code** | **Question** | **Number Code** | **Question** |
| *Demographics* | | | |
| Pt_D1. | Study Participant Code | Pt_D1_F. | Study Participant Code |
| Pt_D2. | What is your current age? | Pt_D2_F. | What is your current age? |
| Pt_D3. | What gender do you consider yourself to be? | Pt_D3_F. | What gender do you consider yourself to be? |
| Pt_D4. | What racial or ethnic group do you consider yourself to belong to? | Pt_D4_F. | What racial or ethnic group do you consider yourself to belong to? |
| Pt_D4a. | Are you Hispanic or Latinx? | Pt_D4_Fa. | Are you Hispanic or Latinx? |
| Pt_D5. | What is the zip code/postal code where you are living now? | - | - |
| Pt_D6. | What is the highest education degree or certificate you have? | - | - |
| Pt_D7. | Which of these best describes your current work situation? | - | - |
| *General Health* | | | |
| Ptgen1. | In general, would you say your health is: | - | - |
| Ptgen2. | How strongly do you agree or disagree with the following statement “There is something that can improve my health”? | - | - |
| Ptgen3. | Health literacy is the degree to which individuals have the ability to obtain, process, and understand basic health information and services needed to make health decisions.  On a scale of 1-10, where 10 is the highest health literacy, in general how health literate do you think you are? | - | - |
| *Previous Adverse Reactions from Medications* | | | |
| Ptrxn1. | Have you ever been hospitalized from a bad reaction to a medication? | - | - |
| Ptrxn2. | Approximately how many times in your life have you had a bad reaction from a medication that required the medication to be stopped? | - | - |
| *Knowledge* | | | |
| Know1. | Doctors can test your genetic makeup to help predict how you will respond to medication. Your genetic makeup may reveal your risk for a bad reaction to a medication or how well a medication will work for you. This type of testing is called pharmacogenetic testing. Prior to today, had you heard of this kind of testing? | - | - |
| Know2. | Prior to today, have you ever had a pharmacogenetic test completed? | - | - |
| Know3. | How strongly do you agree or disagree with the following statement “Pharmacogenetic testing may help my doctor choose better and safer medications for me.” | Know3_F. | How strongly do you agree or disagree with the following statement “Pharmacogenetic testing may help my doctor choose better and safer medications for me.” |
| Know4. | I want to learn more about pharmacogenetic testing. | Know4_F. | I want to learn more about pharmacogenetic testing. |
| Know4a. | What would you like to learn more about? SELECT ALL THAT APPLY: | Know4a_F. | What would you like to learn more about? SELECT ALL THAT APPLY: |
| Know4b. | How would you like to learn more about pharmacogenetic testing? SELECT ALL THAT APPLY: | Know4b_F. | How would you like to learn more about pharmacogenetic testing? SELECT ALL THAT APPLY: |
| Know5. | If I had pharmacogenetic testing, I expect my healthcare provider would know how to use my test results. | Know5_F. | If I had pharmacogenetic testing, I trust my healthcare provider would know how to use my test results. |
| *Attitudes* | | | |
| Att1. | I am curious about how my genetic code affects my response to medications. | Att1_F. | I am curious about how my genetic code affects my response to medications. |
| Att2. | How interested are you in getting pharmacogenetic testing, if it were free? | Att2_F. | How interested are you in getting pharmacogenetic testing, if it were free? |
| Att3. | How strongly do you agree or disagree with the following statement “Pharmacogenetic testing to help with medication selection should be available for those who need it, regardless of cost.” | Att3_F. | How strongly do you agree or disagree with the following statement “Pharmacogenetic testing to help with medication selection should be available for those who need it.” DO NOT CONSIDER THE COST. |
| Att4. | The idea of pharmacogenetic testing worries me. | Att4_F. | The idea of pharmacogenetic testing worries me. |
| Att4a. | What concerns you about pharmacogenetic testing? SELECT ALL THAT APPLY: | Att4a_F. | What concerns you about pharmacogenetic testing? SELECT ALL THAT APPLY: |
| Att5. | It is a good idea to get pharmacogenetic testing to find out how you will respond to a certain medication. | Att5_F. | It is a good idea to get pharmacogenetic testing to find out how you will respond to a certain medication. |
| *Payment* | | | |
| Pay1. | How much do you think pharmacogenetic testing costs? | - | - |
| Pay2. | What is the most you would be willing to pay out of pocket for pharmacogenetic testing? | Pay2_F. | What is the most you would be willing to pay out of pocket for pharmacogenetic testing? |
| *Sharing* | | | |
| Shar1. | If you were to get pharmacogenetic testing, would you share the results with your spouse/partner? | Shar1_F. | Did you share your pharmacogenetic test results with your spouse/partner? |
| Shar2. | If you were to get pharmacogenetic testing, would you share the results with your parents? | Shar2_F. | Did you share your pharmacogenetic test results with your parents? |
| Shar3. | If you were to get pharmacogenetic testing, would you share the results with your children? | Shar3_F. | Did you share your pharmacogenetic test results with your children? |
| Shar4. | If you were to get pharmacogenetic testing, would you share the results with your siblings? | Shar4_F. | Did you share your pharmacogenetic test results with your siblings? |
| Shar6. | If you were to get pharmacogenetic testing, would you share the results with your pharmacist? | Shar6_F. | Did you share your pharmacogenetic test results with your pharmacist? |
| Shar7. | If you were to get pharmacogenetic testing, would you share the results with a friend? | Shar7_F. | Did you share your pharmacogenetic test results with a friend? |
| Shar8. | How would you most prefer to receive your pharmacogenetic test results? | Shar8_F. | How would you most prefer to receive your pharmacogenetic test results? |
| Shar9. | Who would you most prefer to explain your pharmacogenetic test results to you? | Shar9_F. | Who would you most prefer to explain your pharmacogenetic test results to you? |
| - | - | Shar10_F. | Who did you talk with about your decision to have pharmacogenetic testing? SELCT ALL THAT APPLY: |
| *Satisfaction* | | | |
| - | - | Satis1_F. | Based on my experience, I would undergo pharmacogenetic testing for other medications in the future. |
| - | - | Satis2_F. | Based on my experience, I would recommend pharmacogenetic testing to others. |
| - | - | Satis3_F. | I believe I now have a better understanding of pharmacogenetic testing. |
| - | - | Satis4_F. | Which of the following best reflects your feelings about the pharmacogenetic testing that was done? |
| Satis4a. | I am dreading the test (not looking forward to it) | - | - |
| Satis4b. | I am anxious about the test | - | - |
| Satis4c. | I look forward to getting my results | - | - |
| Satis4d. | I am anxious about my results | - | - |
| Satis4e. | I want to know my results | - | - |
| Satis4f. | I care about my results | - | - |
| Satis5. | Which of the following actions do you think will occur as a result of getting pharmacogenetic testing? SELECT ALL THAT APPLY | Satis5_F. | What happened specifically as a result of getting pharmacogenetic testing? SELECT ALL THAT APPLY |

## Table S3

| **Table S3. Pilot Question Responses** | | |
| --- | --- | --- |
| **Questions** | **Number of pilot testers responding** | **Answers*** |
| On a scale of 1-10, where 10 is the most feasible and 1 is the least feasible: | | |
| How feasible will it be to take this survey at the doctor’s office? | 47 | 7.7 **(**2.54) |
| How feasible will it be to take this survey at home? | 48 | 8.5 **(**2.6) |
| How feasible will it be to take this survey at the research office? | 47 | 8.0 **(**2.7) |
| On a scale of 1-10, where 10 is the most easy and 1 is the least easy: | | |
| How easy was the survey to complete? | 47 | 8.5 (1.7) |
| On a scale of 1-10, where 10 is the most understandable and 1 is the least understandable: | | |
| How well were the questions written to ensure patients, like yourself, will understand? | 46 | 8.3 **(**2.0) |
| On a scale of 1-10, where 10 is filled with medical jargon and 1 has no medical jargon: | | |
| Quantify the amount of “medical jargon” present in the survey questions. | 46 | 3.9 **(**2.8) |
| Other questions: | | |
| Approximately, how many minutes did the pilot survey take you to complete? | 45 | 9.6 (6.1) |
| How appropriate is the length of this survey? n (%) | 48 |  |
| Too long |  | 14 (29) |
| Just right |  | 34 (71) |
| Too short |  | 0 (0) |

*Answers to the questions are summarized as mean (SD) unless otherwise specified

## Figure S1

**Figure S1.** A pie chart of the themes of feedback received. A total of 133 comments were received from 25 pilot testers. Each comment was categorized into one of five themes. Numbers are expressed as number, (%)

## Figure S2

**Figure S2.** The themes of feedback that resulted in modification of the survey instrument. Of the 133 comments received, 47 resulted in a modification of the survey question or answer.

## Figure S3


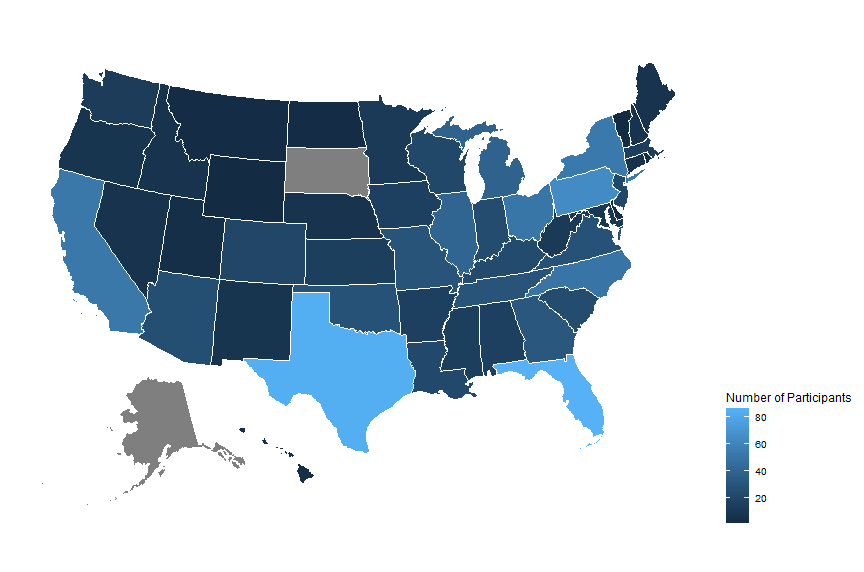


**Figure S3.** Distribution of respondents across the United States in final cohort (n=1060). Shading depicts count of participants where the lighter the shading the greater number of participants.

# Medically Underserved Nationwide Survey Instrument

Exploring individual preferences for pharmacogenetic and pre-emptive pharmacogenetic testing

Start of Block: Screening Questions

S1 Are you 18 years of age or older?

- Yes (1)
- No (2)

| Page Break |  |
| --- | --- |

S2 Do you currently live in the United States?

- Yes (1)
- No (2)

| Page Break |  |
| --- | --- |

S3 Is your approximate average yearly household income $42,000 or less?

- Yes (1)
- No (2)

End of Block: Screening Questions

Start of Block: Introduction and Waiver of Documentation of Informed Consent

**Title of Project:** Exploring individual preferences for pharmacogenetic testing

 **IRB Study Number:** IRB202200571

**Principal Investigator:** Julio Duarte, PharmD, PhD, Assistant Professor
 Department of Pharmacotherapy and Translational Research
 University of Florida College of Pharmacy

1. **Purpose of the Study**: The purpose of this study is to assess the attitudes, perceptions, and willingness to pay for pharmacogenetic testing—testing of how your genes (the information or features passed from your parents to you) might affect the way you respond to certain medicines.

 2. **Procedures to be followed:** You will be asked to answer between 39-42 questions in this survey.

 3. **Duration:** The survey should take about 15 minutes to complete.

 4. **Research Benefits:** There is no direct benefit to you for being in this study. There may be a benefit to others depending on the results of this study.

 5. **Research Risks:** There are no risks or discomfort anticipated for this study.

 6. **Statement of Confidentiality:** Your participation in this research is confidential. The survey does not ask for any sensitive information or any information that could identify who you are, other than your zip code. Data will be stored on secured, password-protected computers and the link between your zip code and responses will be removed at the end of the study.

 7. **Right to Ask Questions:** Please contact Julio Duarte at juliod@cop.ufl.edu or (352) 273-8132 with questions or concerns about this study.

 8. **Payment for participation:** No compensation for participation in this study will be provided by the study investigators.

 9. **Voluntary Participation:** Your decision to be in this study is voluntary. You may stop taking the survey at any time.

 If you have any questions about your rights as a research participant, you can call the UF Institutional Review Board at (352) 273-9600.

 Clicking next and continuing with the survey implies that you have read the information on this page and consent to take part in the research.

End of Block: Introduction and Waiver of Documentation of Informed Consent

Start of Block: Demographics

|  |
| --- |

Q1 What is your current age?

________________________________________________________________

Q2 What gender do you identify as?

- Male (1)
- Female (2)
- Other (3)

| Page Break |  |
| --- | --- |

Q3 What racial group do you primarily identify as?

- Caucasian/White (1)
- Black/African American (2)
- Asian (3)
- American Indian/Alaskan Native (4)
- Pacific Islander/Native Hawaiian (5)
- Mixed Race (6)
- Another Race (7)

Q4 Do you identify as Hispanic, Latino, or Latinx?

- Yes (1)
- No (2)
- I don't know (3)

|  |
| --- |

Q5 What is the 5-digit zip code/postal code where you are living now?

________________________________________________________________

End of Block: Demographics

Start of Block: Socioeconomic

Q6 What is the highest education level you have achieved?

- Elementary or junior high (1)
- GED (2)
- High school diploma (3)
- Vocational tech diploma (4)
- Associate degree (5)
- Bachelor's degree (6)
- Master's degree (7)
- Doctorate or professional degree (8)

Q7 Do you currently, or have you in the past, worked in the health field or biomedical science field?

- Yes (1)
- No (2)
- I don't know (3)

| Page Break |  |
| --- | --- |

Q8 Health literacy is how much a person can find, process, and understand basic health information needed to make health decisions. How would you rank your health literacy?

|  | Not health literate | Somewhat health literate | Very health literate |
| --- | --- | --- | --- |

| Please slide the slide bar to rank your level of health literacy () | 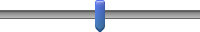 |
| --- | --- |

| Page Break |  |
| --- | --- |

Q9 Which of these options best describes your current work situation?

- Working full-time (35 hours or more a week) (1)
- Working part-time (less than 35 hours a week) (2)
- Full-time homemaker (3)
- Not able to work due to disability (4)
- Retired (5)
- Unemployed or laid off and looking for work (6)
- Something else (7)

Q10 How many people currently live in your household (including yourself)?

- 1 (1)
- 2 (2)
- 3 (3)
- 4 (4)
- 5 (5)
- 6 or more (6)

| Page Break |  |
| --- | --- |

Q11 What is your approximate average yearly household income?

- Less than $17,500 (1)
- $17,500 to $26,500 (2)
- $26,600 to $35,500 (3)
- $35,600 to $42,000 (4)
- Greater than $42,000 (5)

Q12 How would you describe the financial situation in your household right now?

- After paying the bills, I still have enough money to purchase additional things I want (1)
- After paying the bills, I rarely have enough money to purchase additional things I want (2)
- I only have enough to pay the bills because I cut back on other things (3)
- I have trouble paying the bills no matter what I do (4)

End of Block: Socioeconomic

Start of Block: Pre-emptive Pharmacogenetic Explanation

E2 Pharmacogenetic testing provides information about how your genes (the information or features passed from your parents to you) might affect the way you respond to certain medicines. The results can help in understanding if your genes make you likely to be more or less sensitive to certain medicines, meaning you might need a different dose.


Pharmacogenetic results can also provide information about which medicines might work better for you and which medicines to avoid because they might either not work well or could cause unwanted side effects. Because your genes stay the same throughout your lifetime, pharmacogenetic tests only have to be completed once and the results can be used for the rest of your life. 


There are two common ways pharmacogenetic testing can be completed: 


1. Reactively: If your doctor is considering prescribing you a medication, he/she can order a certain pharmacogenetic test for that medication to help make a decision. 


2. Preemptively: You can get a single test that provides results for many medications before they are needed. Then, when your doctor is deciding which medication to prescribe you, the results are already in your medical records.

End of Block: Pre-emptive Pharmacogenetic Explanation

Start of Block: Previous Experience with Pharmacogenetics

Q13 Prior to today, had you heard of pharmacogenetic testing?

- Yes (1)
- No (2)

Q14 To your knowledge, have you ever had a pharmacogenetic test ordered by a healthcare professional (doctor, nurse, pharmacist, etc.)?

- Yes (1)
- No (2)
- I don't know (3)

End of Block: Previous Experience with Pharmacogenetics

Start of Block: Cost of Testing

Q15 How interested are you in receiving pharmacogenetic testing if there were no cost to you?

- Very interested (1)
- Moderately interested (2)
- Somewhat interested (3)
- Not at all interested (4)

Q16 What do you think the current out of pocket cost for pharmacogenetic testing is?

- Nothing ($0) (1)
- $1-24 (2)
- $25-49 (3)
- $50-99 (4)
- $100-199 (5)
- $200-399 (6)
- $400-599 (7)
- $600-999 (8)
- $1,000 or more (9)

| Page Break |  |
| --- | --- |

Q17 What is the most you would be willing to pay out of pocket for pharmacogenetic testing?

- Nothing ($0) (1)
- $1-24 (2)
- $25-49 (3)
- $50-99 (4)
- $100-199 (5)
- $200-399 (6)
- $400-599 (7)
- $600-999 (8)
- $1,000 or more (9)

Q18 Would you be willing to pay $100 out of pocket for a one-time pre-emptive pharmacogenetic testing panel? This single test would contain results for many medications and your test results would already be available in your medical records when your doctor makes a decision about prescribing you a medication.

- Yes (1)
- No (2)

Display This Question:

If Would you be willing to pay $100 out of pocket for a one-time pre-emptive pharmacogenetic testing... = No

Q19a Would you be willing to pay $50 out of pocket for a one time pre-emptive pharmacogenetic testing panel? This single test would contain results for many medications and your test results would already be available in your medical records when your doctor makes a decision about prescribing you a medication.

- Yes (1)
- No (2)

Display This Question:

If Would you be willing to pay $100 out of pocket for a one-time pre-emptive pharmacogenetic testing... = Yes

Q19b Would you be willing to pay $200 out of pocket for a one time pre-emptive pharmacogenetic testing panel? This single test would contain results for many medications and your test results would already be available in your medical records when your doctor makes a decision about prescribing you a medication.

- Yes (1)
- No (2)

Display This Question:

If Would you be willing to pay $200 out of pocket for a one time pre-emptive pharmacogenetic testing... = Yes

Q20b Would you be willing to pay $300 out of pocket for a one time pre-emptive pharmacogenetic testing panel? This single test would contain results for many medications and your test results would already be available in your medical records when your doctor makes a decision about prescribing you a medication.

- Yes (1)
- No (2)

End of Block: Cost of Testing

Start of Block: DCE Explanation

E3 This section describes different factors that you might consider when deciding about getting a pharmacogenetic test. Below are characteristics of the tests and their explanations. 


Please read these carefully — in the next section of the survey you will be asked to make choices based on how important these factors are to you.
 
Recommended by your doctor: Whether or not getting the pharmacogenetic test is recommended by your doctor 
 
Wait (turnaround) time: How long you and your doctor would have to wait before getting the test results
 
Number of actionable results in your lifetime: How many times in your life that the results from your test will be used to help prescribe or adjust your medications
 
Benefit of the test: The greatest benefit you would receive from the test:


 "Avoid a minor side effect" means that the results lead your doctor to prescribe a different medication that avoided a minor side effect, such as nausea. 


"Avoid a major side effect" means the results helped to avoid a major side effect that might put you in the hospital. 


"Find a medicine likely to work for a minor health problem" means the results helped your doctor choose a medicine being prescribed for a minor medical problem. 


"Find a medicine likely to work for a major health problem" is like the previous option but this time the medicine is being prescribed for a major health problem or disease that might be life-threatening
 
Out-of-pocket cost of testing: How much you would pay out-of-pocket for the test

| Page Break |  |
| --- | --- |

E4 During the next 9 questions, we will present two types of pharmacogenetic tests. There will be some similarities and differences between the two tests. We want you to imagine that you are deciding which of the two tests you wish to get. 


To help the accuracy of this section, even though you will not actually be paying for the test please consider the actual dollar amounts of the tests that are presented to you. Remember that if you spend the money on the test, that amount of money could no longer be used for other expenses such as rent, utilities, or grocery bills.


Before choosing, please carefully consider all the test characteristics. Then, please choose the test you would prefer. If you would choose to have both tests, please select the one that you think would be the best for you. If you would choose to not have either of these tests, please select the test that is closest to a test that you would choose.

End of Block: DCE Explanation

Start of Block: DCE Block1


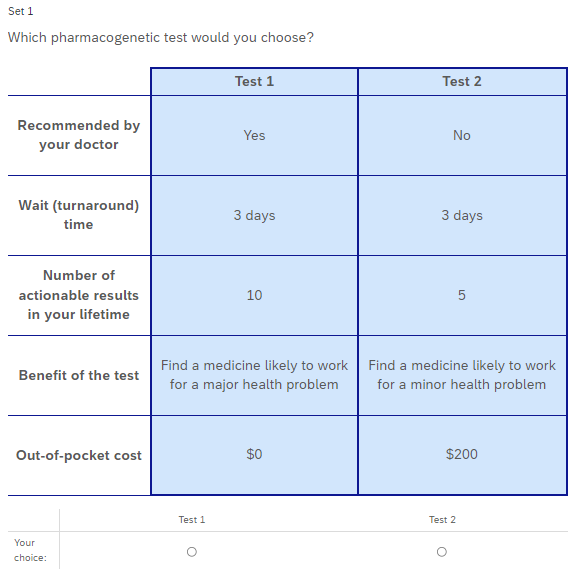


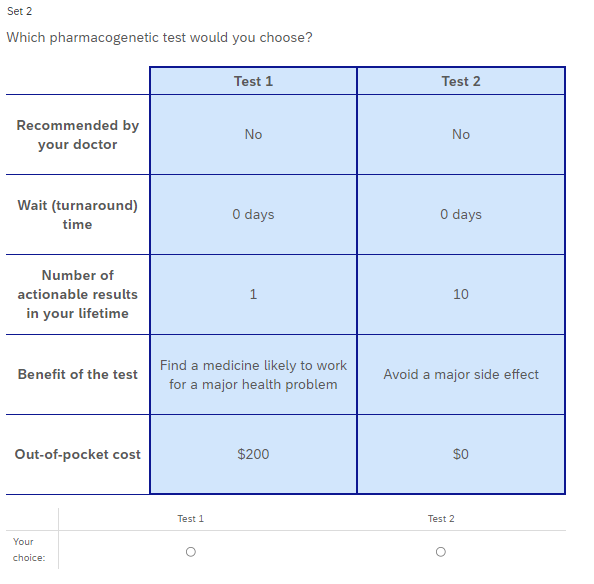


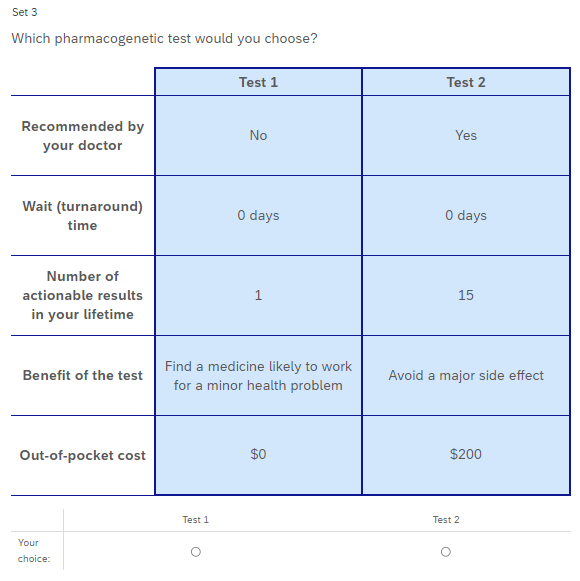


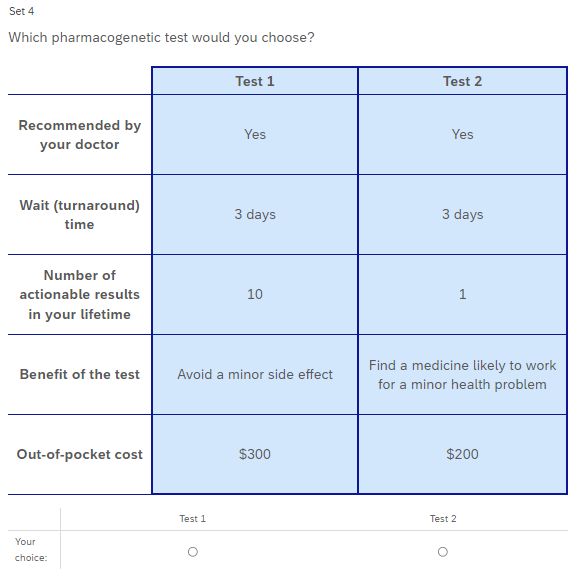


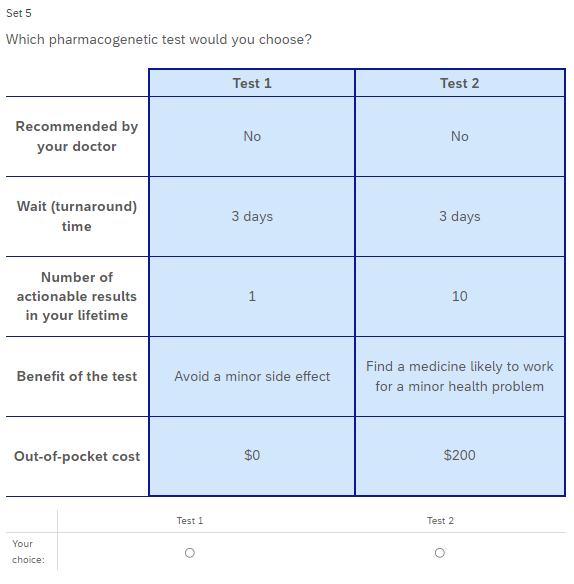


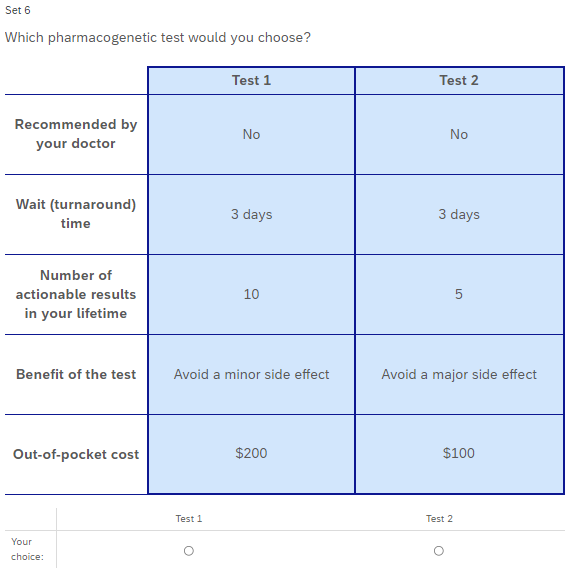


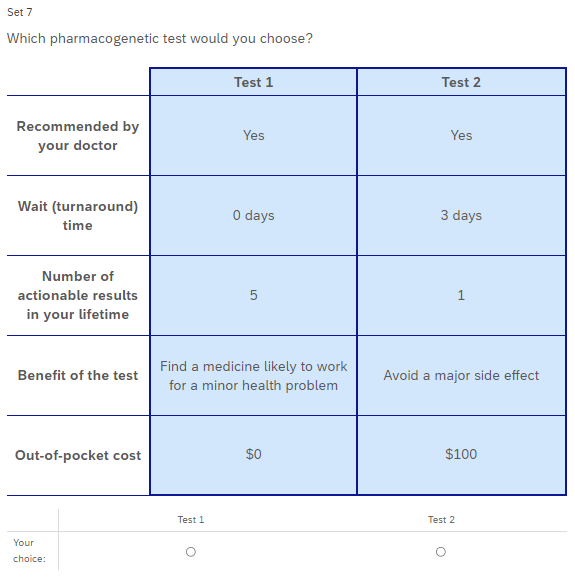


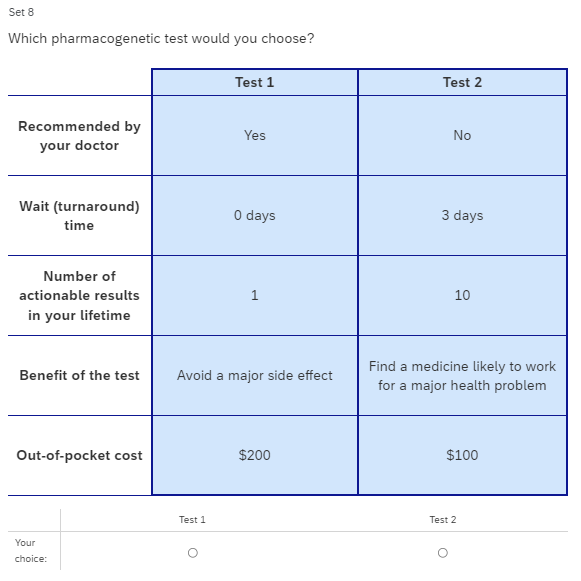


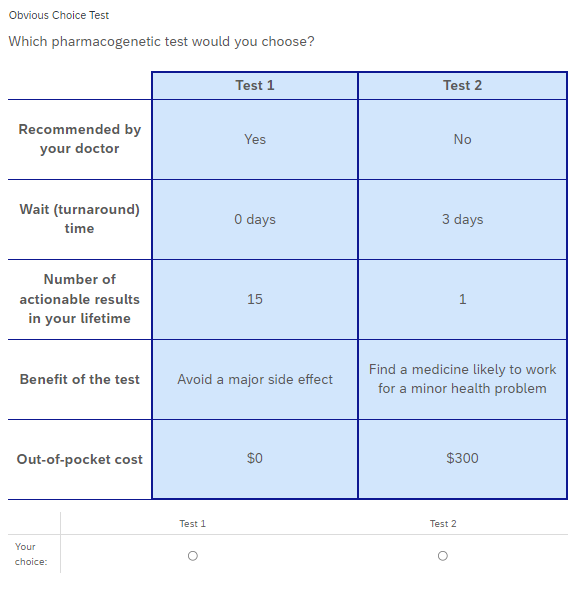


End of Block: DCE Block1

Start of Block: DCE Block 2


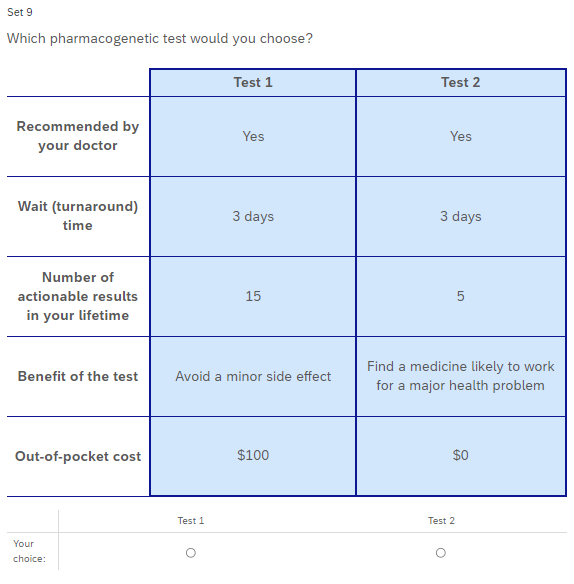


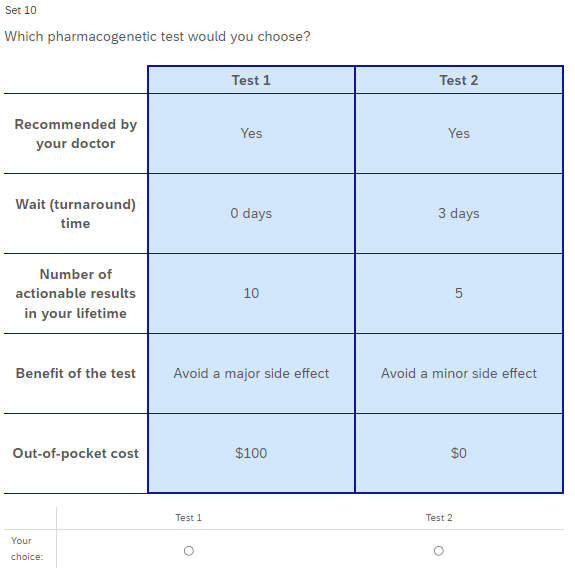


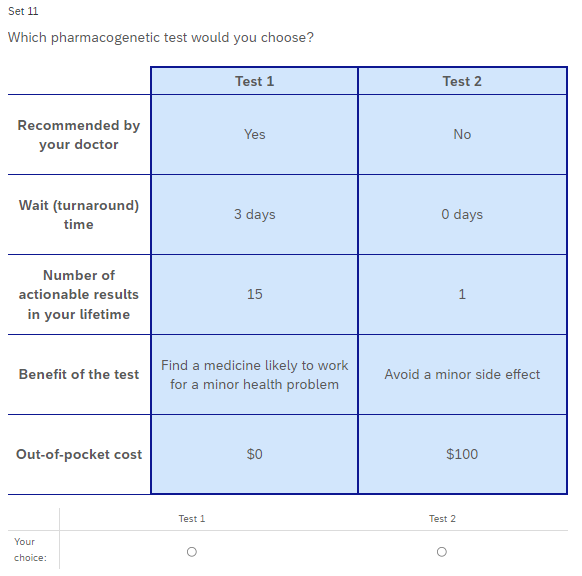


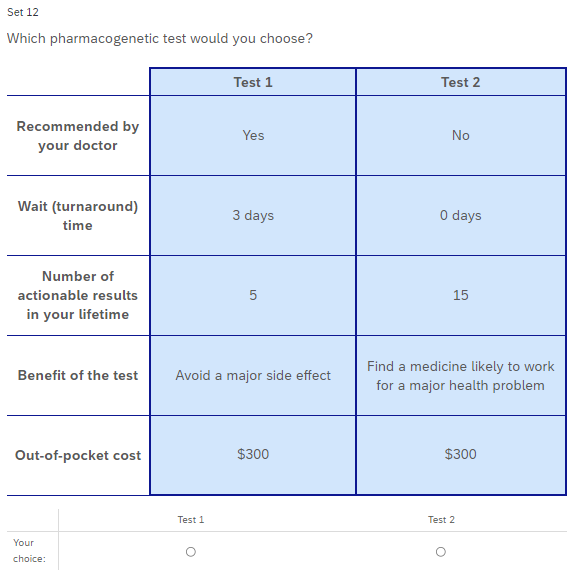


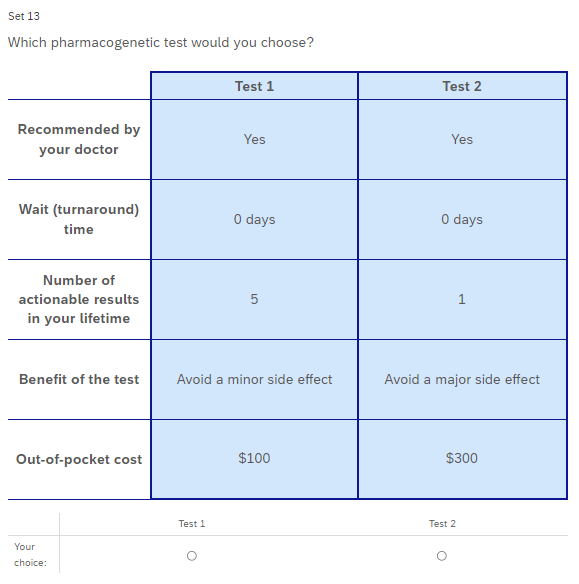


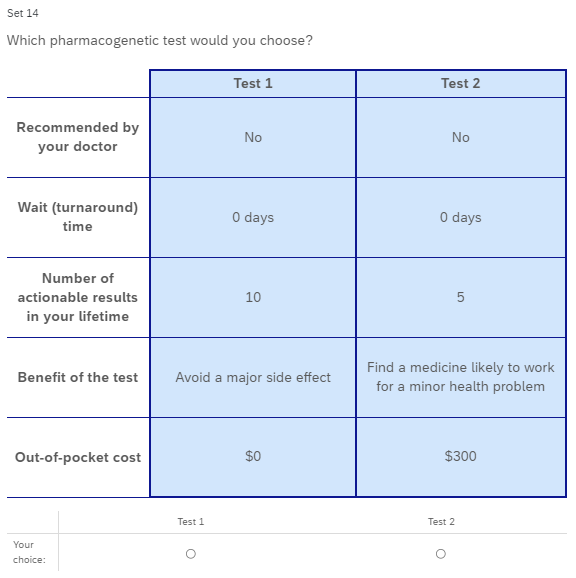


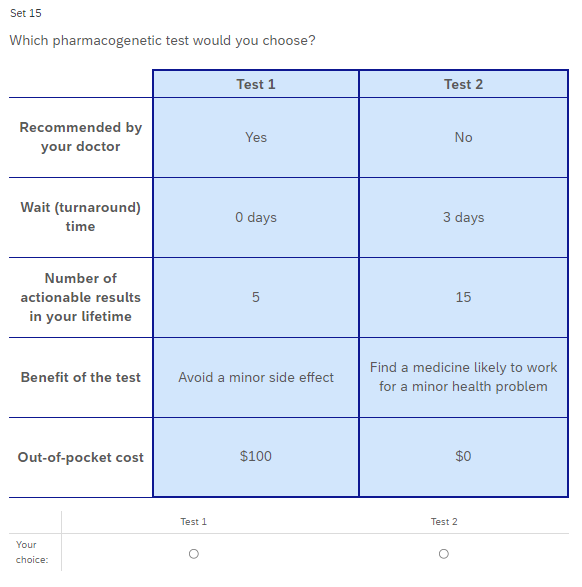


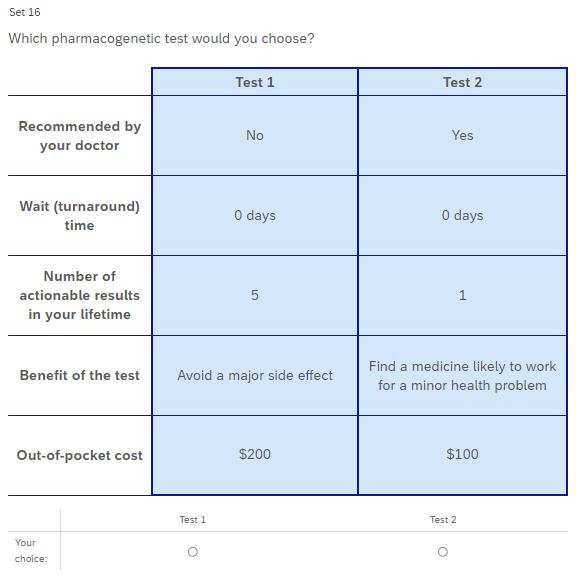


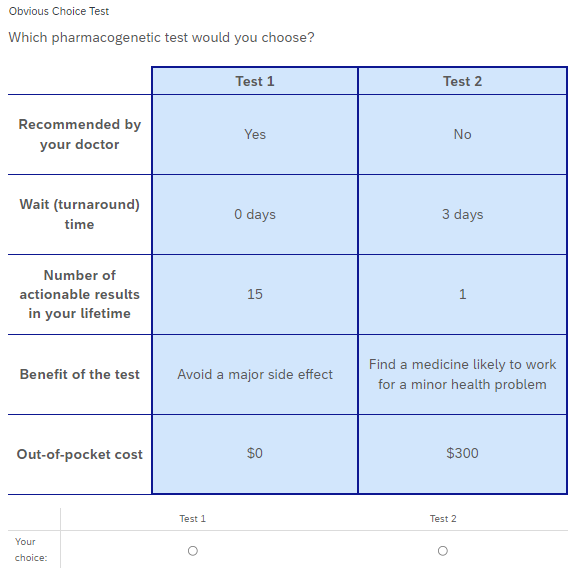


End of Block: DCE Block 2

Start of Block: Health Status

Q30 In general, would you say your health is:

- Excellent (1)
- Very good (2)
- Good (3)
- Fair (4)
- Poor (5)

Q31 How many times have you seen a healthcare provider (doctor, nurse, pharmacist etc.) in the past 12 months?

- 0 (1)
- 1 (2)
- 2 (3)
- 3 (4)
- 4 (5)
- 5 or more times (6)

| Page Break |  |
| --- | --- |

Q32 How strongly do you agree or disagree with the following statement "There is something available that can improve my health"?

- Strongly agree (1)
- Agree (2)
- Neither agree nor disagree (3)
- Disagree (4)
- Strongly disagree (5)
- Not applicable (6)

Q33 Have you ever had a bad reaction to a medication that required the medication to be stopped?

- Yes (1)
- No (2)
- I don't know (4)

| Page Break |  |
| --- | --- |

Display This Question:

If Have you ever had a bad reaction to a medication that required the medication to be stopped? = Yes

Q33a Did the bad reaction to the medication require you to be hospitalized?

- Yes (1)
- No (2)

| Page Break |  |
| --- | --- |

Q34 Do you have health insurance?

- Yes (1)
- No (2)

Display This Question:

If Do you have health insurance? = Yes

Q34a What type of health insurance do you have?

- Commercial (includes insurance through your job or insurance that you purchased) (1)
- Medicare (2)
- Medicaid (3)
- Other government provided (VA benefits, military, etc.) (4)

End of Block: Health Status

Start of Block: Attitudes and Opinions on Pharmacogenetic Testing

Q35 For the following statements, please rate how strongly you agree or disagree.

|  | Strongly agree (1) | Agree (2) | Neither agree nor disagree (3) | Disagree (4) | Strongly disagree (5) |
| --- | --- | --- | --- | --- | --- |
| If I had pharmacogenetic testing, I expect that my healthcare provider would know how to use my test results. (1) |  |  |  |  |  |
| Pharmacogenetic testing may help my doctor choose better and safer medications for me. (2) |  |  |  |  |  |
| Pharmacogenetic testing to help with medication selection should be available for those who need it, regardless of cost. (3) |  |  |  |  |  |
| It is a good idea to receive pharmacogenetic testing to estimate how you will respond to certain medications. (4) |  |  |  |  |  |

| Page Break |  |
| --- | --- |

Q36 For the following statements, please rate how strongly you agree or disagree.

|  | Strongly agree (1) | Agree (2) | Neither agree nor disagree (3) | Disagree (4) | Strongly disagree (5) |
| --- | --- | --- | --- | --- | --- |
| I want to learn more about pharmacogenetic testing. (1) |  |  |  |  |  |
| I would be disappointed if my current medications are not impacted by my pharmacogenetic test results (2) |  |  |  |  |  |
| I am curious about how my genetic code affects my response to medications. (3) |  |  |  |  |  |
| The idea of pharmacogenetic testing worries me (4) |  |  |  |  |  |
| I would be disappointed if my pharmacogenetic results were different than what I expected. (5) |  |  |  |  |  |

| Page Break |  |
| --- | --- |

Q37 What concerns, if any, do you have about pharmacogenetic testing? (Please check all that apply)

- The results could affect my health insurance (1)
- The results could affect other insurance (life, disability, etc.) (2)
- The results could affect my employment (3)
- The results could affect my family (4)
- The results could tell me I am at risk for a certain disease (5)
- The test will cost me money (6)
- The results will not improve my care (7)
- Another concern (8)
- None of these things concern me (9)

| Page Break |  |
| --- | --- |

Q38 If you were to receive pharmacogenetic testing, would you share the results with...

|  | Yes (1) | No (2) | Not Applicable (3) |
| --- | --- | --- | --- |
| your spouse/partner? (1) |  |  |  |
| your parents? (2) |  |  |  |
| your children? (3) |  |  |  |
| your siblings? (4) |  |  |  |
| your other family members? (5) |  |  |  |
| your pharmacist? (6) |  |  |  |

| Page Break |  |
| --- | --- |

Q39 If you received pharmacogenetic testing, how would you most prefer to receive your test results?

- Phone (1)
- Email (2)
- Electronic health records or online health portal (3)
- Mail (4)
- In person (5)
- No preference (6)

| Page Break |  |
| --- | --- |

Q40 Who would you most prefer to explain your pharmacogenetic test results to you?

- A doctor (1)
- A nurse (2)
- A genetic counselor (3)
- A pharmacist (4)
- Someone else (5)
- No preference (6)

End of Block: Attitudes and Opinions on Pharmacogenetic Testing
